# Improving nurses’ readiness for evidence-based practice in critical care units: results of an information literacy training program

**DOI:** 10.1186/s12912-021-00599-y

**Published:** 2021-05-18

**Authors:** Jamileh Farokhzadian, Somayeh Jouparinejad, Farhad Fatehi, Fatemeh Falahati-Marvast

**Affiliations:** 1 Nursing Research Center, Kerman University of Medical Sciences, Kerman, Iran; 2 Department of Community Health Nursing, Razi Faculty of Nursing and Midwifery, Kerman University of Medical Sciences, Kerman, Iran; 3 Health in Disasters and Emergencies Research Center, Institute for Futures Studies in Health, Kerman University of Medical Sciences, Kerman, Iran; 4 School of Psychological Sciences, Monash University, Melbourne, Australia; 5 Centre for Online Health, The University of Queensland, Brisbane, Australia; 6 Health Information Sciences Department, Faculty of Management and Medical Information Sciences, Kerman University of Medical Sciences, Kerman, Iran

**Keywords:** Nursing informatics, Information literacy, Evidence-based practice, Training program, Nursing education

## Abstract

**Background:**

One of the most important prerequisites for nurses’ readiness to implement Evidence-Based Practice (EBP) is to improve their information literacy skills. This study aimed to evaluate the impact of a training program on nurses’ information literacy skills for EBP in critical care units.

**Methods:**

In this interventional study, 60 nurses working in critical care units of hospitals affiliated to Kerman University of Medical Sciences were randomly assigned into the intervention or control groups. The intervention group was provided with information literacy training in three eight-hour sessions over 3 weeks. Data were collected using demographic and information literacy skills for EBP questionnaires before and 1 month after the intervention.

**Results:**

At baseline, the intervention and control groups were similar in terms of demographic characteristics and information literacy skills for EBP. The training program significantly improved all dimensions of information literacy skills of the nurses in the intervention group, including the use of different information resources (3.43 ± 0.48, *p* < 0.001), information searching skills and the use of different search features (3.85 ± 0.67, *p* < 0.001), knowledge about search operators (3.74 ± 0.14, *p* < 0.001), and selection of more appropriate search statement (*x*^*2*^ = 50.63, *p* = 0.001) compared with the control group.

**Conclusions:**

Nurses can learn EBP skills and apply research findings in their nursing practice in order to provide high-quality, safe nursing care in clinical settings. Practical workshops and regular training courses are effective interventional strategies to equip nurses with information literacy skills so that they can apply these skills to their future nursing practice.

## Background

The Institute of Medicine (IOM) has recommended that Evidence-Based Practice (EBP) be used in 90% of clinical decisions by 2020 [1]. EBP has become a popular buzzword in the healthcare industry [1, 2]. This concept is defined as a problem-solving process in clinical decision making, which combines best research evidence with clinical expertise, patient preference, and clinical guidelines. EBP is required to improve quality of care, patient outcomes, and cost effectiveness of care [2, 3]. It is a gold standard that provides a framework for delivery of safe and compassionate care. EBP is not only the use of research results but also includes all aspects of nursing knowledge, attitudes, skills and self-efficacy. It is considered as an essential skill for nurses to use the best scientific evidence when designing and implementing healthcare programs as well as when applying the available research evidence in decision-making process [2].

Critical care nurses are responsible for the assessment of patients and provision of care in critical care units. Critical care nurses need expertise and evidence to recognize clinical changes and prevent complications in patients. EBP should be applied to provide better care for patients in critical care units [4]. Policymakers expect nurses to make decisions based on the most recent evidence [5]. Therefore, nurses must be equipped with information literacy skills to obtain research findings and up-to-date information [6, 7]. Information literacy is fundamental for successful implementation of EBP, and nurses should learn and improve their search and retrieval skills in order to obtain best evidence and information for providing sympathetic, safe and ethical care [7–11].

Despite the emphasis on EBP being applied in nurses’ daily practice, a significant number of nurses and other clinicians have not get involved in EBP and are not fully aware of the concept of EBP. Nurses are unprepared to implement EBP due to a lack of information literacy skills in information searching and retrieval. Therefore, there is a gap between nurses’ ability and implementation of EBP [2, 3, 12–14]. According to the nursing and medical literatures, nurses are facing difficulties with EBP and their most difficult task is to find the best evidence, identify the right sources, use optimal search methods, and critically appraise the evidence in general [15]. In recent years, health care policymakers have focused on the EBP as a means of improving health services and quality of care [16].

Several studies have been conducted to determine the significance of information literacy in the implementation of EBP. The New York University Division of Nursing, for example, used components of information literacy in core courses of a master’s program to provide nursing professionals with the skills [17]. One study addressed the importance of searching for, evaluating, synthesizing and applying documented information and found that more than 80% of the nurses did not receive any training related to EBP [10]. A number of other studies investigated the importance of information literacy and the need for education programs to enhance nursing search and retrieval skills [18–20]. Moreover, nurses’ readiness for EBP was measured by predictors of EBP, including nurse’s informational needs and skills in using EBP, attitudes, knowledge and workplace culture in order to identify desired interventions to make EBP more practical [21, 22]. Concerning the importance of decision-making in critical care units, this study investigated the impact of a training program on nurses’ information literacy skills for EBP in critical care units.

The following hypotheses were tested:

- Hypothesis 1: Intervention group’s mean scores on the use of different information resources would improve after a training program compared with the control group.
- Hypothesis 2: Intervention group’s mean scores of information searching skills and use of different search features would increase after a training program compared with the control group.
- Hypothesis 3: Intervention group’s mean scores of knowledge about search operators would increase after a training program compared with the control group.
- Hypothesis 4: Intervention group’s mean scores of frequency of selecting more appropriate search strategy would increase after a training program compared with the control group.

## Methods

### Study design and settings

This interventional study with pretest-posttest design and two intervention and control groups was conducted from March to April 2019. Nurses working in critical care units (ICUs, CCUs, and Dialysis) were selected from three educational hospitals affiliated with Kerman University of Medical Sciences in the southeast of Iran. All methods were performed in accordance with the relevant guidelines and regulations of this university.

### Participants and sampling

All nurses (*N* = 330) working in critical care units at the time of data collection were included in the study. A sample size of 27 participants was calculated for each group (54 participants for the two groups) using a previous study and the sample size formula. By taking into account α = 0.05, test power of 80%, and large effect size (Cohen d = 0.7) and 10% dropout probability, 60 nurses were recruited in the study using the stratified random sampling method (30 nurses in each group). Three separate lists were created from nurses working in critical care settings of the three hospitals. Owing to the fact that the number of nurses in the three hospitals was almost equal, the random number table was used to select 20 nurses equally from each hospital (10 nurses for the intervention group and 10 nurses for the control group). Finally, these nurses were divided into the intervention (*n* = 30) and control groups (*n* = 30).

The inclusion criteria were nurses with a bachelor’s degree or higher, as well as at least 6 months of work experience in critical care units. Participants who missed more than one session and did not complete questionnaires were excluded from the study.

### Instruments

Two questionnaires were used in this study. The first one concerned the nurses’ demographic information, including gender, age, work experience, organizational position, type of shift, level of education, marital status, and history of participation in research and information literacy courses (Table 2). The second questionnaire assessed information literacy skills for EBP, indicating EBP readiness. This questionnaire was developed by a professional team of faculty members and nurses [10, 23]. The information literacy skills for EBP questionnaire comprised two sections. The first section covered the use of different information resources — print, electronic and human with 19 items on a 5-point Likert scale ranging from “never” to “always”. The second section collected data on information searching skills and the use of different search features of online databases and web search engines such as subject headings and search operators, and included 10 items on a 5-point Likert scale ranging from “never” to “always”. Nurses’ knowledge about Boolean/Connectors (‘OR’, ‘AND’, ‘NOT’ or ‘AND NOT’) and Proximity (e.g. W/nn; PRE/nn) operators was assessed using 4 items with yes (one score), no (zero score), and not sure options (zero score). Finally, to assess the nurses’ database searching skills and actual skills in developing an effective search statement, they were given a hypothetical searching topic (Effect of cigarettes on lung Cancer) along with five possible search statements on MEDLINE. They were asked to choose the most appropriate search statement.

The cross-cultural adaptation, validity and reliability (α = 0.87) of this questionnaire has been established by Farokhzadian et al. [24].

### Data collection

The self-reported questionnaires were distributed among nurses of the intervention and control groups in the pretest (before workshop) and posttest stages (one month after the workshop). Except for the intervention group that received additional material derived from workshop and the control group that received no educational program during this period, all participants completed questionnaires simultaneously and attended the routine or traditional programs in hospitals. In other words, the two study groups were subjected to the same job descriptions. However, in order to improve internal validity of the study, researchers monitored the study conditions thoroughly to ensure that the intervention and control groups were similar in all aspects, except attendance at the training program. All participants completed the survey.

### Intervention procedure

The training workshops were conducted in three eight-hour sessions over 3 weeks. The intervention group was divided into two groups to increase the members’ chances of participating in the workshop. Using lectures, questions and answers, slide presentations, hands-on and online exercises, homework, and educational CDs, one Ph.D. nurse and three experts in the field of medical informatics conducted the educational course. They have entered interactive computer-based search engines with the participants during the training.

Table 1 shows the content presented in this workshop.

**Table 1:** Topics presented in the workshop

**Session 1** EBP fundamentals • What is EBP? • The significance of EBP in health care • Required skills for EBP activities • Different resources for EBP • Identification of clinical issues/problems • Translation of a clinical issue/problem into a well-formulated clinical question by using the PICO format • Conduction of online searches • Critical appraisal of research articles by using checklist • Extraction of a general insight from strengths and weaknesses of research studies • Implementation of EBP in the nursing practice	
**Session 2** Information literacy skills • A brief introduction of hardware • Explanation of operating system, software and electronic files such as PDF and Text • The teaching of types of web browsers and their features • Introduction of databases such as PubMed, Scopus, CINAHL, and SID	
**Session 3** Information literacy skills • Review of the educational contents of the previous sessions • Teaching of search strategies in databases such as PubMed and Scopus •Explanation of the online database features ○ The ways to subscribe to and receive free articles ○ Simple and advanced searches ○ Limited search (publication date, full-text availability, article type and etc.) ○ Field search (keywords, MeSH, abstract, and etc.) ○ Boolean operators (AND, OR, NOT) • The participants practiced what they learned simultaneously. • Final exercise in a clinical scenario ○ Introducing a title “Intubate Patient Care” ○ Searching in the PubMed database with related keywords and providing search results ○ Answering participants’ questions ○ Using several keywords and presenting the related articles in this session ○ Practically using the contents of prior sessions for retrieving scientific evidences ○ Sending articles obtained by nurses to the professors	

### Statistical analysis

The data were analyzed in SPSS 21 by using descriptive statistics (frequency, percentage, mean and standard deviation) and inferential statistics (independent samples *t*-test, paired *t*-test, McNemar-test, chi square, and Fisher’s exact test). The Kolmogorov-Smirnov test showed that the data were normally distributed. The significance level was considered ≤0.05.

## Results

### Demographic and professional information

All nurses (intervention and control groups) participated in the study and completed questionnaire (response rate = 100%). The nurses were asked to provide their demographic and professional characteristics. No significant difference was found between the intervention and control groups in terms of demographic and professional information (Table 2).

**Table 2:** Comparison of demographic and professional information of nurses in the intervention and control groups

Variables	Groups	Intervention		Control		Statistic and *P*
		n	%	n	%	
Gender	Male	5	16.70	5	16.70	*χ2 =* 0.003 *P* = 0.99
	Female	25	83.30	25	83.30	
Age groups	20–40	25	83.30	24	80	*χ2 =* 0.15 *P* = 0.64
	> 40	5	16.70	6	20	
Marital status	Single	9	29.10	8	26.70	*χ2 =* 1.06 *P* = 0.58
	Married	21	70.90	22	73.30	
Work experience (year)	< 5	8	25.80	12	40	Fisher’s exact test = 3.86
	5–10	4	12.90	4	13.30	*P* = 0.42
	11–15	12	41.90	6	20	
	16–20	4	12.90	4	13.30	
	> 21	2	6.50	4	13.30	
Work experience in critical care units (year)	< 5	7	25	11	36.60	Fisher’s exact test = 3.86 *P* = 0.42
	5–10	9	28.60	8	26.60	
	11–15	11	35.70	9	28.60	
	16–20	2	7.10	1	3.60	
	> 21	1	3.60	1	3.60	
Work position	Head nurse	0	0	3	10	Fisher’s exact test = 3.26 *P* = 0.7
	Nurse	30	100	27	90	
Shift work	Fixed	1	3.20	2	6.70	Fisher’s exact test = 0.38 *P* = 0.53
	Rotated	29	96.80	28	93.30	
Participation in research courses	Yes	1	3.20	5	16.70	Fisher’s exact test = 3.10 *P* = 0.07
	No	29	96.80	25	83.30	
Participation in information literacy courses	Yes	2	6.50	5	16.70	Fisher’s exact test = 1.56 *P* = 0.21
	No	28	93.50	25	83.30	

### Use of different information resources

No significant difference was found between the intervention (2.66 ± 0.70) and control (2.67 ± 0.66) groups in the pretest mean scores of use of different information resources (*t* = 0.10, *P* = 0.92). However, a significant difference was observed between the intervention (3.43 ± 0.48) and control (2.76 ± 0.60) groups in terms of use of different information resources in the posttest (*t* = 4.90, *p* < 0.001), showing that the training program significantly improved the use of different information resources in the intervention group. In addition, results showed that the training program had the highest impact on the use of different electronic resources (1.11) and the lowest impact on the use of human resources (0.26) (Table 3).

**Table 3:** Comparison of mean scores of the use of different information resources in intervention and control groups

Information resources	Groups	Pre test	Post test	Mean difference	Statistic t* and *p*
		*M* ± SD	*M* ± SD		
Printed	Intervention	2.98 ± 0.73	3.62 ± 0.57	0.64	*t* = −6.54 ***p*** **< 0.001**
	Control	3.04 ± 0.88	2.99 ± 0.73	−0.05	*t* = −1.14 *p* = 0.26
	Statistic t** and *p*	*t* = −0.99 *p* = 0.32	*t* = 3.55 ***p*** **= 0.001**		
Electronic	Intervention	2.39 ± 0.87	3.50 ± 0.97	1.11	*t* = −8.49 ***p*** **= 0.01**
	Control	2.48 ± 0.88	2.43 ± 0.83	−0.05	*t* = 2.28 ***p*** **= 0.03**
	Statistic t** and *p*	*t* = −0.40 *p* = 0.69	*t* = 5.11 ***p*** **< 0.001**		
Human	Intervention	2.86 ± 0.83	3.12 ± 0.51	0.26	*t* = −2.71 ***p*** **= 0.01**
	Control	2.89 ± 0.54	2.35 ± 0.52	−0.54	*t* = 0.06 *p* = 1.00
	Statistic t** and *p*	*t* = −0.16 *p* = 0.87	*t* = 1.98 ***p*** **= 0.05**		
Total	Intervention	2.66 ± 0.70	3.43 ± 0.48	0.77	*t* = −7.15 ***p*** **< 0.001**
	Control	2.67 ± 0.66	2.76 ± 0.60	0.09	*t* = 0.08 *p* = 1.00
	Statistic t** and *p*	*t* = −0.10 *p* = 0.92	*t* = 4.90 ***p*** **< 0.001**		

*Paired t-test

**Independent t-test

As shown in Table 3, the use of different information resources in the control group had no significant difference in the pretest and posttest.

### Information searching skills and use of different search features

No significant difference was found between the intervention (2.06 ± 0.76) and control (2.19 ± 0.83) groups in the pretest mean scores of the information searching skills and the use of different search features (*t* = − 0.59, *P* = 0.55). However, a significant difference was observed between the intervention (3.85 ± 0.67) and control (1.93 ± 0.70) groups in terms of information searching skills and the use of different search features in posttest (*t* = 10.92, *p* < 0.001), showing that the training program significantly improved information searching skills and the use of different search features in the intervention group. In addition, result showed that the searching skills and use of different search features in the control group had no significant difference at pretest and posttest (Table 4).

**Table 4:** Comparison of mean scores of the information searching skills and use of different search features

Variable	Groups	Pre test	Post test	Mean difference	Statistic t* and *p*
		*M* ± SD	*M* ± SD		
Information searching skills	Intervention	2.06 ± 0.76	3.85 ± 0.67	1.79	*t* = −8.38 ***p*** **< 0.001**
	Control	2.19 ± 0.83	1.93 ± 0.70	−0.26	*t* = −1.24 *p* = 0.25
	Statistic t** and *p*	*t* = −0.59 *p* = 0.55	*t* = 10.92 ***p*** **< 0.001**		

*Paired t-test

**Independent t-test

### Knowledge about search operators

No significant difference was found between the intervention (0.61 ± 0.23) and control (0.56 ± 0.21) groups in the pretest mean scores of knowledge about search operators (*t* = − 0.14, *p* = 0.88). However, a significant difference was observed between the intervention (3.74 ± 0.14) and control (0.33 ± 0.12) groups in terms of knowledge about search operators in the posttest (*t* = 17.37, *p* < 0.001), showing that the training program improved significantly knowledge about search operators in the intervention group. In addition, result showed that the control group’s knowledge about search operators had no significant difference in the pretest and posttest (Table 5).

**Table 5:** Comparison of mean scores of knowledge about search operators in intervention and control groups

Operators	Groups	Pre test	Post test	Mean difference	Statistic t* and *p*
		*M* ± SD	*M* ± SD		
AND	Intervention	0.16 ± 0.06	0.96 ± 0.03	0.80	*t* = −9.40 ***p*** **< 0.001**
	Control	0.20 ± 0.08	0.13 ± 0.06	−0.07	*t* = 1.00 *p* = 0.32
	Statistic t** and *p*	*t* = −44 *p* = 0.66	*t* = 11.77 ***p < 0.001***		
OR	Intervention	0.19 ± 0.07	0.96 ± 0.03	0.77	*t* = −8.66 ***p*** **< 0.001**
	Control	0.21 ± 0.08	0.13 ± 0.06	−0.08	*t* = 1.00 *p* = 0.32
	Statistic t** and *p*	*t* = −0.21 *p* = 0.83	*t* = 11.77 ***p < 0.001***		
NOT	Intervention	0.12 ± 0.06	0.93 ± 0.04	0.80	*t* = −9.40 ***p*** **< 0.001**
	Control	0.12 ± 0.06	0.06 ± 0.04	−0.06	*t* = 1.00 *p* = 0.32
	Statistic t** and *p*	*t* = 0.04 *p* = 0.96	*t* = 13.47 ***p*** **< 0.001**		
Proximity operator	Intervention	0.12 ± 0.06	0.87 ± 0.06	0.74	*t* = −8.03 ***p*** **< 0.001**
	Control	0.10 ± 0.03	0.08 ± 0.05	−0.02	*t* = 0.02 *p* = 0.80
	Statistic t** and *p*	*t* = 2.10 ***p*** **= 0.04**	*t* = 14.23 ***p*** **< 0.001**		
Total	Intervention	0.61 ± 0.23	3.74 ± 0.14	3.12	*t* = −9.86 ***p*** **< 0.001**
	Control	0.56 ± 0.21	0.33 ± 0.12	−0.23	*t* = 0.01 *p* = 0.84
	Statistic t** and *p*	*t* = −0.14 *p* = 0.88	*t* = 17.37 ***p*** **< 0.001**		

*Paired t-test

**Independent t-test

### Assessment of developing search strategy

In the pretest phase, no significant difference was found between the intervention (25.80%, *n* = 8) and control (3.2%, *n* = 1) groups in frequency of selecting more appropriate search statement (*x*^*2*^ = 6.36, *P* = 0.01). In addition, frequency of selecting more appropriate search statement increased significantly in the intervention group (93.50%, *n* = 29) compared with the control (3.2%, *n* = 1) group in the posttest (*x*^*2*^ = 50.63, *p* = 0.001). In addition, frequency of selecting more appropriate search statement in the control group had no significant difference in the pretest and posttest (Table 6).

**Table 6:** Comparison of frequency of selecting more appropriate search statement in intervention and control groups

Variable	Groups	Pre test	Post test	Statistic * and *p*
		n(%)	n(%)	
Selecting more appropriate search statement (item 4)	Intervention	8(25.80)	29(93.50)	**0.001**
	Control	1(3.20)	1(3.20)	1.00
	Statistic ** and *p*	6.36 **0.01**	50.63 **0.001**	

*McNemar-test

**chi square-test

## Discussion

The present study investigated the impact of a training program on nurse’s information literacy skills for EBP in the critical care units of three hospitals affiliated to an Iranian university. The findings showed that the training program significantly improved the use of different information resources in the intervention group. Furthermore, the nurses in intervention group sought information more from electronic resources than from human and printed resources at posttest. In agreement with our findings, Tannery et al. [25] designed a pre/post-intervention study and provided access to a collection of online knowledge-based resources. After the intervention, nurses began to use various information resources as well as electronic resources instead of colleagues and print textbooks or journals. In a 4-year longitudinal study, Weng et al. [26] examined information seeking behaviors for EBP in the physicians and nurses through implementing an EBM multifaceted program that included access to websites, databases, libraries, and workshops. They found that the use of print resources remained unchanged while the use of electronic resources increased. Fiander et al. [27] in a review study reported that the use of electronic information increased in the intervention groups participating in training programs. The present study finding disagrees with the cross-sectional study conducted by Thiel et al. [21], which found that the majority of nurses obtained informational needs from peers or colleagues rather than journals, books and electronic databases. According to Patelarou et al. [22], nurses use electronic databases infrequently, with young nurses and university graduates being more likely to use them. A descriptive study was done by Farokhzadian et al. [24] who showed that nurses used different information resources, including more human and printed resources than electronic ones to search information. One of the main reasons for nurses’ poor use of electronic resources is their unfamiliarity with online databases and insufficient search skills.

The results showed that our training program improved significantly information searching skills and the use of different search features in intervention group at posttest. Kratochvíl [28] also showed positive impact of an information literacy course on students in the post-test stage, which improved their knowledge about search skills such as subject heading, defining keywords and wildcards. Carlock and Anderson [29] assessed information literacy skills of student nurses in two groups after teaching them the instructions and holding sessions. Then, for further investigation, one group was followed up while the other was not. Students in the follow-up group improved their search skills, while students in the non-follow-up group made more mistakes in their searches. Using valid and reliable nurse’s readiness tool for EBP with four specific domains including “EBP-attitude”, “EBP-knowledge”, “informational needs” and “workplace culture” [30], Patelarou et al. [22]reported an average level of nurse’s skill to conduct a search in CINAHL or MEDLINE databases based on informational needs domain. They also found “EBP knowledge” domain was positively correlated with both “informational needs” and “workplace culture” domains, implying that a nurse can learn EBP knowledge by developing necessary skills in an EBP-friendly workplace.

The findings indicated that the educational program improved significantly knowledge about search operators and frequency of selecting more appropriate search statement in the intervention group at the posttest. Therefore, the educational program improved significantly the database searching skills and actual skills in developing an effective search statement in nurses of the intervention group.

Thiel and Ghosh [21] discovered that the perceived nurse’s EBP knowledge was at a moderate level and significantly correlated with the nurse’s education level and years of nursing experiences. They described perceived EBP knowledge as different from actual knowledge and suggested that providing various resources for EBP teaching–learning plans was a helpful tool to evaluate actual knowledge of EBP over time. In addition, Kratochvíl [28] showed that students’ knowledge about search statement and Boolean operators increased after educational intervention. In a randomized controlled trial, Brettle et al. [31] evaluated the effectiveness of an online information literacy tutorial and a face-to-face session for teaching information literacy skills to nurses. The searching skills, including developing search strategy and using search operators, improved after intervention and remained unchanged in both methods 1 month later, but no improvement in any of the methods was observed after one month. El-sayed et al. [32] showed improvement in the search of information resources and search strategy among master nursing students through a training program. Ruzafa-Martínez et al. [33] conducted a 15-week educational course on EBP for the undergraduate nursing students, covering topics such as search strategies, Boolean operators, and limit function, and showed that knowledge and skill for EBP improved in the interventional group. In their pretest-posttest research, Hsieh et al. [34] employed an EBP program for search strategy and electronic literature search and evaluated learning outcomes before, immediately after, and 3 months after the intervention. They found that knowledge and skill increased immediately after the intervention, but then decreased in the final follow-up. Therefore, continuous training is essential for the stability and durability of the learned skills through follow-up courses in the previous studies. In contrast, Farokhzadian et al. [24] reported in a descriptive study that most of the nurses knew nothing or very little about information literacy skills like advanced searching techniques, Boolean and proximity operators, search features such as truncations, wildcards, MeSH terms, and search limits. Majid et al. [23]investigated nurses’ literature searching skills and discovered that they used basic search features and that less than one-quarter were familiar with Boolean and proximity operators. They also reported that nurses who had previously attended EBP training programs had more knowledge than those who had not. Hirt et al. [35] conducted a review study of educational interventions on health-related literature searching skills. They indicated that participants in the included studies were students and physicians from various health professions. The study findings were divided into two categories: search strategy development and database searching skills. Some interventional studies included in this review reported significant improvements in the development of search strategies and database searching skills, while others showed no changes in these two categories. Therefore, nurses must have sufficient knowledge to improve their information literacy skills in order to retrieve information related to clinical practice, improve the quality of care and make better decisions.

Diverse and efficient methods are recommended to empower nurses in information literacy, including integrating computer and search skills training program into the nursing curriculum in different semesters, ongoing education through holding practical workshops and sessions, following seeking behaviors among nurses and presenting feedback to them, easy access to online databases in clinical wards, updating nurses’ information about changes in search features and user interfaces of online database such as launch of the “new PubMed”, continuous encouragement to turn EBP into a routine activity, development of an information center and educational spaces equipped with IT facilities, and provision of 24/7 online trainings. Further research on the efficacy of these interventions is also suggested by using controlled study designs and long-term follow-up. According to the findings of a review study [35], few educational interventional studies on search skills have been conducted in the last 10 years. Therefore, more emphasis must be placed on nurses' education.

### Limitations

We did not evaluate the effectiveness of follow-up courses in this study because nurses working in the critical care units did not have enough time and motivation to participate in these courses due to heavy shift works. This study investigated the effect of training program on nurses in critical care units and similar interventions could be performed on nurses in other wards to ensure the results are generalizable.

## Conclusions

The results of this study show that the training program can effectively improve nurses’ information literacy skills. Information literacy is one of the key components of EBP for identifying and evaluating available scientific evidence. Nurses need search skills in order to find and use evidence in their nursing practice and provide positive patient outcomes. Therefore, it is critical to develop educational programs to help nurses improve their information literacy skills. Our findings provide health planners and policymakers with the opportunity to design an educational model that is effective, practical, and continuous in order to strengthen the nurses’ skills and make EBP more practical in the clinical setting. Extensive developments in EBP will eventually lead to improvement in healthcare and health services quality.

## Data Availability

The datasets used and/or analyzed during the current study are available from the corresponding author on reasonable request.

## Abbreviations

EBP: Evidence-Based Practice
PICO: Population/Patient or Problem, Intervention/Exposure, Comparison, Outcome
CINAHL: Cumulated Index to Nursing and Allied Health Literature
MeSH: Medical Subject Headings
SID: Scientific Information Database
